# Application of energy and nutrient reductions to a mixed-cereal diet without added inorganic phosphate, supplemented with a novel phytase alone or with a xylanase–β-glucanase combination, achieved a production benefit in pigs from wean to finish

**DOI:** 10.1093/tas/txaf060

**Published:** 2025-05-04

**Authors:** Deepak E Velayudhan, Yueming Dersjant-Li, Ester Vinyeta, Georg Dusel

**Affiliations:** Danisco Animal Nutrition & Health (IFF), Willem Einthovenstraat 4, 2342 BH Oegstgeest, the Netherlands; Danisco Animal Nutrition & Health (IFF), Willem Einthovenstraat 4, 2342 BH Oegstgeest, the Netherlands; Danisco Animal Nutrition & Health (IFF), Willem Einthovenstraat 4, 2342 BH Oegstgeest, the Netherlands; University of Applied Sciences Bingen, Berlinstrasse 109, 55411 Bingen am Rhein, Germany

**Keywords:** bacterial 6-phytase, β-glucanase, growth performance, pigs, xylanase

## Abstract

This experiment tested the hypothesis that supplementation of a nutrient- and energy-reduced mixed-cereal diet with phytase, xylanase and β-glucanase, over an entire wean-to-finish growth cycle, would result in growth performance outcomes that were not different from those achieved by pigs fed an unsupplemented, nutritionally-adequate diet. A total of 192 weaned pigs [DanBred × Pi, initial body weight (BW) 7.2 ± 0.4 kg] were assigned to 48 floor pens [4 pigs/pen (2 male, 2 female), 12 pens/treatment], in a completely randomized design. Diets included: 1) a nutritionally adequate wheat, corn and barley-based positive control (**PC**); 2) a negative control (**NC**) based on the PC but without added inorganic P, reduced in Ca, net energy (**NE**), digestible amino acids (**AA**) and Na, supplemented with PhyG at 1,000, 1,000, 750, 500 and 500 FTU/kg in starter I (7 to 11 kg BW), starter II (11 to 25 kg BW), grower I (25 to 55 kg BW), grower II (55 to 85 kg BW) and finisher (85 to 115 kg BW) phases, respectively (NC1 + PhyG low); 3) NC1 further reduced in Ca, digestible AA and NE, (by ≤ 0.03 percentage points, ≤ 0.01 percentage points and ≤ 9 kcal/kg, respectively) supplemented with PhyG at 2,000, 2,000, 1,000, 750 and 750 FTU/kg per phase (NC2 + PhyG high), and; 4) as 2) but further reduced in NE and digestible AA (by 26 to 33 kcal/kg and ≤ 0.01 percentage points, respectively), supplemented with 2,440 XU/kg xylanase and 304 U/kg β-glucanase (NC3 + PhyG low + XB). For the overall period, growth performance (all measures) was maintained in the enzyme-supplemented treatments to a level not different from the PC, whereas in starter II and grower I, BW was increased (+ 1.82 and + 5.11 kg/pig, respectively; *P* < 0.05) and gain:feed was increased (*P* < 0.05) in NC3 + PhyG low + XB, compared with the PC. Total estimated feed costs per kilogram BW gain (BWG) were lower (*P* < 0.05) in NC3 + PhyG low + XB (-0.05 € or 7.3%) and the carbon footprint of production was reduced in NC2 + PhyG high and NC3 + PhyG low + XB compared with the PC (by 128 and 145 g CO_2_ equivalents per kilogram of BWG, respectively, equivalent to reductions of 6.0% and 6.8%; *P* < 0.05). These results confirm the appropriateness of the applied energy and nutrient reductions for PhyG and PhyG with xylanase–β-glucanase in a mixed-cereal diet from wean to finish and highlight a potential feed cost saving and environmental sustainability benefit of the application.

## Teaser

This experiment has confirmed the appropriateness of prescribed dietary energy and nutrient reductions for use with a phytase enzyme at two dose levels, with or without a xylanase–β-glucanase combination, in a mixed-cereal diet without added inorganic phosphate, in pigs from wean to finish. The results will be of value in the future application of the tested enzymes in least-cost diets for pigs.

## INTRODUCTION

In Europe, commercial pig diets are often formulated with wheat, rye or barley, either as the primary cereal or as an addition to corn. Industrial co-products such as wheat bran and distillers’ dried grains with solubles are also frequently added. Wheat, rye and barley are high in starch and therefore rich sources of energy, but are also higher in fiber and associated non-starch polysaccharides (**NSP**) than corn (~12%, 17% and up to 20% fiber in wheat, barley and rye, respectively, vs. ~7% in corn; Botticella et al., 2021). Although dietary fiber is important for digestion and intestinal health, particularly in young pigs, high dietary fiber with associated high soluble NSP content can reduce nutrient digestibility and impair growth (Moeser and van Kempen, 2002; Agyekum and Nyachoti, 2017). Industrial co-products are also high in fiber and NSP (Rosenfelder et al., 2013; Jaworski et al., 2015). Furthermore, wheat, barley, rye and co-product ingredients also tend to be higher in phytate (inositol hexakisphosphate, IP_6_) than corn (Selle and Ravindran, 2007). Phytate is a rich source of P but one that is poorly utilized in pigs due to an insufficiency in endogenous phytate-degrading enzymes.

In such complex diets, supplemental phytate- and fiber-degrading enzymes can be added to improve nutrient and energy availability and utilization. Microbial phytase is commonly added to increase the availability of P from plant-derived phytate (Humer et al., 2015) but can also improve the digestibility of Ca, energy, AA and Na (Espinosa et al., 2021, 2022; Velayudhan et al., 2022). These effects enable reductions in the nutrient and energy content of the diet to be made to account for the expected contribution of the enzymes. As nutrient digestibility responses to exogenous phytase are highly dose-dependent (Dersjant-Li and Dusel, 2019; Dersjant-Li et al., 2020) the dose can be carefully titrated in diets of differing composition to match the substrate content, giving consideration to the change in animal nutrient requirements by age.

In fibrous cereal ingredients, xylans, arabinoxylans and β-glucans are among the major NSP present. Rye and wheat are rich in soluble arabinoxylans (Bach Knudsen, 2014) and barley is rich in β-glucans. In diets containing these cereals and fibrous co-products, supplemental xylanase and β-glucanase can be effective for improving nutrient and energy availability. For example, a xylanase–β-glucanase combination dosed at 610 or 2,440 xylanase units (**XU**)/kg) and 76 or 304 units β-glucanase units (**U**)/kg increased the availability and utilization of energy for growth and weight gain in growing pigs fed a mixed-cereal diet containing co-products (Kiarie et al., 2012). As phytase and carbohydrases operate with different modes of action on substrates that reside in different parts of the plant cell structure (phytate inside cells and NSP within plant cell walls), these enzymes may have complementary effects in improving energy and nutrient availability.

A recently marketed commercial bacterial 6-phytase was demonstrated to be efficacious for increasing mineral digestibility and utilization in weaned pigs fed nutrient- and energy-reduced mixed-cereal diets (Velayudhan et al., 2025). This same phytase can also increase the digestibility of AA and energy in grower pig diets (Velayudhan et al., 2021; Espinosa et al., 2022). However, to the authors’ knowledge, the combination of the phytase with xylanase and β-glucanase, which may allow further reductions in the dietary content of digestible AA and energy, has not been previously tested in pigs during an entire wean-to-finish growth period. The hypothesis tested in the present study was that supplementation of a nutrient- and energy-reduced mixed-cereal diet with the phytase alone, or in combination with xylanase and β-glucanase, would result in growth performance outcomes over an entire wean-to-finish period that were not different from those achieved by pigs fed an unsupplemented, nutritionally-adequate diet. We also evaluated whether the nutrient and energy reductions afforded by the enzymes would reduce the total estimated feed costs and carbon footprint (**CFP**) of the diet per kilogram of liveweight gain.

## MATERIALS AND METHODS

The experiment was carried out at the University of Applied Sciences Bingen, Germany. It was conducted in accordance with animal welfare practice guidelines in Germany.

### Pigs, Housing and Experimental Design

A total of 192 (DanBred × Pi) mixed-sex pigs (initial body weight 7.2 ± 0.4 kg) weaned at 28 d of age were assigned on the day of weaning to 48 floor-pens (each pen 1.50 × 1.75 m) on the basis of initial body weight (**BW**) so that each pen contained pigs of approximately equal initial BW. There were 4 pigs per pen (2 castrated males, 2 females) and 12 pens per treatment, in a completely randomized design. Pens were located in environmentally controlled animal rooms in which the temperature was maintained at 30 °C during the first week of the experiment and then gradually reduced to 24 °C by week 6. The lighting regime was light-dark (**LD**) 16:8 h.

At the start of the grower phase (after 46 d on trial, ~27 kg BW), pigs were moved to the grower-finisher barn. During this phase pigs were housed in 96 pens with 24 pens per treatment and 2 pigs per pen (1 castrated male and 1 female; each set of 4 pen mates from nursery being split into 2 and reassigned to pens (with 2 pigs per pen), based on BW to minimize BW variation between pens. Pen size during grower and finisher phase was 2.60 × 1.10 m.

### Treatment Diets

There were 4 treatment diets, each formulated in 5 phases (starter I, 7 to 11 kg BW; starter II, 11 to 25 kg BW; grower I, 25 to 55 kg BW; grower II, 55 to 85 kg BW and finisher, 85 to 115 kg BW). Treatment details are given in Table 1. Diets were based on wheat, corn, barley, rye and soybean meal, with added rapeseed meal (Tables 2–4). The positive control (**PC**) was formulated to meet the nutritional requirements of pigs in Germany, in accordance with GfE, 2006. Three negative control (**NC**) diets were formulated. Negative control 1 (NC1) and 2 (NC2) were formulated without added inorganic P and were reduced in Ca, NE, digestible AA and Na vs. PC, according to the manufacturer’s recommended nutrient matrix for PhyG phytase based on the actual dose level applied to each treatment during each phase (Table 1). Negative control 3 (NC3) was formulated with similar ingredient and nutrient content as NC1 but with additional reductions in NE and digestible AA applied across phases to account for the expected contribution of the added xylanase–β-glucanase combination. The NC1 diet was supplemented with a novel consensus bacterial 6-phytase variant [Axtra PHY GOLD (herein termed PhyG), Danisco Animal Nutrition & Health (IFF), the Netherlands] produced in *Trichoderma reesei,* at a “low” dose, comprising 1,000, 1,000, 750, 500 and 500 FTU/kg during starter I, starter II, grower I, grower II and finisher phase, respectively (NC1 + PhyG low). The NC2 was supplemented with PhyG at a ‘high’ dose, comprising 2,000, 2,000, 1,000, 750 and 750 FTU/kg, during starter I, starter II, grower I, grower II and finisher phase, respectively (NC2 + PhyG high), and the NC3 was supplemented with PhyG as in NC1 + PhyG low and in addition with a xylanase-β-glucanase combination [Axtra XB, Danisco Animal Nutrition & Health (IFF), the Netherlands] to provide xylanase at 2,440 XU/kg and β-glucanase at 304 U/kg (NC3 + PhyG low + XB). The xylanase-β-glucanase comprised of a 1,4-β-xylanase (EC 3.2.1.8) produced in *T. reesei* and an endo-1,3(4)-β-glucanase (EC 3.2.1.6) produced in *T. reesei*). An unsupplemented NC diet was not included due to potential health issues caused by the extent of the nutrient reductions applied to the NC diets. Enzymes were pre-mixed with a 10 kg portion of the appropriate basal diet before addition to the main batch. All diets were then thoroughly mixed to ensure a homogeneous distribution of the enzymes. Diets were fed to pigs in pellet form (3 to 4 mm pellet; pelleting temperature < 75 °C). Experimental diets and water were provided ad libitum for the duration of the experiment (140 d).

**Table 1.** Treatment details

Treatment No.	Treatment description	PhyG, FTU/kg					Xylanase, XU/kg	β-glucanase, U/kg
		Starter I (7 to 11 kg)	Starter II (11 to 25 kg)	Grower I (25 to 55 kg)	Grower II (55 to 85 kg)	Finisher (85 to 115 kg)	All phases	All phases
1	PC	None					None	None
2	NC1 + PhyG low	1,000	1,000	750	500	500	None	None
3	NC2 + PhyG high	2,000	2,000	1,000	750	750	None	None
4	NC3 + PhyG low + XB	1,000	1,000	750	500	500	2,440	304

**Table 2.** Ingredient composition (as-fed basis) and calculated nutrient content of the experimental diets^1^ during starter I and II phase (exclusive of supplemental enzymes)

Phase	Starter I (7 to 11 kg BW)				Starter II (11 to 25 kg BW)			
	PC	NC1 + PhyG low	NC2 + PhyG high	NC3 + PhyG low + XB	PC	NC1 + PhyG low	NC2 + PhyG high	NC3 + PhyG low + XB
Ingredient, % unless otherwise stated								
Wheat	20.63	26.07	26.32	26.71	27.86	31.67	31.95	32.42
Corn	22.00	22.00	22.00	22.00	18.00	18.00	18.00	18.00
Barley	10.00	10.00	10.00	10.00	15.00	15.00	15.00	15.00
Rye	5.00	5.00	5.00	5.00	7.50	7.50	7.50	7.50
Soybean meal (46.0% CP)	20.00	17.50	17.40	17.50	21.50	19.50	19.40	19.40
Fermented soy protein	5.00	5.00	5.00	5.00	-	-	-	-
Rapeseed meal	3.00	3.00	3.00	3.00	5.00	5.00	5.00	5.00
Whey powder	5.00	4.00	4.00	4.00	-	-	-	-
Lactose	3.00	3.00	3.00	3.00	-	-	-	-
Soybean oil	3.00	1.90	1.80	1.30	1.90	0.90	0.80	0.30
L-lysine HCl	0.50	0.49	0.48	0.47	0.50	0.49	0.48	0.48
DL-methionine	0.20	0.18	0.18	0.17	0.12	0.10	0.10	0.09
L-threonine	0.15	0.13	0.13	0.13	0.15	0.14	0.14	0.13
L-tryptophan	0.03	0.02	0.02	0.02	0.02	-	-	-
L-valine	0.04	0.03	0.03	0.02	-	-	-	-
Calcium carbonate	1.05	0.90	0.88	0.90	0.95	0.90	0.87	0.90
Monocalcium phosphate	0.60	-	-	-	0.70	-	-	-
Sodium chloride	0.30	0.28	0.26	0.28	0.30	0.28	0.26	0.28
Vitamin-mineral premix^2^	0.50	0.50	0.50	0.50	0.50	0.50	0.50	0.50
Calculated nutrients, % unless otherwise stated								
Net energy, kcal/kg	2,451	2,395	2,389	2,356	2,451	2,395	2,388	2,356
Ash	5.56	5.04	5.03	5.00	5.31	4.84	4.80	4.83
Crude protein	19.02	18.80	18.83	18.78	19.21	18.91	18.88	18.90
Crude lipid	4.96	3.98	3.90	3.44	5.02	4.22	3.97	3.80
Crude fiber	3.24	3.28	3.26	3.33	3.20	3.28	3.31	3.33
Starch	37.7	37.8	37.9	38.2	40.9	41.0	41.2	41.4
Lysine (SID)	1.36 (1.24)	1.33 (1.21)	1.32 (1.21)	1.31 (1.20)	1.30 (1.14)	1.28 (1.12)	1.28 (1.12)	1.27 (1.11)
Methionine (SID)	0.46 (0.44)	0.44 (0.42)	0.44 (0.42)	0.44 (0.42)	0.41 (0.39)	0.40 (0.38)	0.40 (0.38)	0.40 (0.38)
Methionine + cysteine (SID)	0.81 (0.74)	0.80 (0.72)	0.80 (0.71)	0.79 (0.71)	0.75 (0.66)	0.74 (0.64)	0.73 (0.63)	0.73 (0.63)
Threonine (SID)	0.84 (0.74)	0.82 (0.72)	0.82 (0.71)	0.82(0.71)	0.82 (0.69)	0.79 (0.67)	0.79 (0.66)	0.79 (0.66)
Tryptophan (SID)	0.25 (0.22)	0.24 (0.20)	0.24 (0.20)	0.24 (0.20)	0.24 (0.21)	0.22 (0.20)	0.21 (0.19)	0.21 (0.19)
Valine (SID)	0.92 (0.80)	0.90 (0.77)	0.90 (0.77)	0.90 (0.77)	0.82 (0.72)	0.80 (0.70)	0.79 (0.69)	0.80 (0.70)
Isoleucine (SID)	0.82 (0.72)	0.80 (0.70)	0.79 (0.69)	0.80 (0.70)	0.76 (0.66)	0.74 (0.63)	0.73 (0.63)	0.74 (0.63)
Leucine (SID)	1.45 (1.29)	1.41 (1.25)	1.41 (1.24)	1.41 (1.25)	1.36 (1.20)	1.32 (1.16)	1.32 (1.16)	1.32 (1.16)
Calcium	0.75	0.58	0.55	0.58	0.73	0.58	0.57	0.58
Phosphorus	0.55	0.42	0.41	0.42	0.55	0.39	0.39	0.39
ATTD of phosphorus	0.27	0.17	0.17	0.17	0.27	0.14	0.14	0.14
Sodium	0.20	0.18	0.18	0.18	0.20	0.20	0.20	0.20
Phytate phosphorus	0.22	-	-	-	0.26	-	-	-

^1^Treatment details are given in Table 1.

^2^Provided per kilogram of diet: 15,750 IU of Vitamin A; 1,800 IU of Vitamin D3; 135 mg of Vitamin E; 1.80 mg of Vitamin K3; 225 mcg of Vitamin H (Biotin); 3.00 mg of Folic acid; 3.15 mg of Vitamin B1; 6.30 mg of Vitamin B2; 5.40 mg of Vitamin B6; 50.00 μg of Vitamin B12; 31.50 mg of Niacinamide (niacin); 19.73 mg of Calcium-D-pantothenic acid; 148.07 mg of Betaine-HCL; 500.00 mg Choline chloride; 67.50 mg of Fe (sulfate); 67.50 mg of Fe-glycine (org.); 93.38 mg of Cu (Cu-chelate), 45.00 mg of Mn (oxide); 67.50 mg of Zn (oxide); 45.00 mg of Zn-glycine (org.); 0.90 mg of I; 0.32 mg of Sodium selenate; and 0.04 mg of Se-methionine.

ATTD, apparent total tract digestible; NE, net energy; SID, standardized ileal digestible.

**Table 3.** Ingredient composition (as-fed basis) and calculated nutrient content of the experimental diets^1^ during grower I and II phase (exclusive of supplemental enzymes)

	Grower I (25 to 55 kg BW)				Grower II (55 to 85 kg BW)			
	PC	NC1 + PhyG low	NC2 + PhyG high	NC3 + PhyG low + XB	PC	NC1 + PhyG low	NC2 + PhyG high	NC3 + PhyG low + XB
Ingredient, % as is, unless otherwise stated								
Wheat	23.00	26.23	26.64	27.30	27.80	31.45	31.87	32.27
Corn	15.00	15.00	15.00	15.00	12.70	12.70	12.70	12.70
Barley	14.50	14.50	14.50	14.50	17.50	17.50	17.50	17.50
Rye	10.00	10.00	10.00	10.00	12.00	12.00	12.00	12.00
Wheat bran	6.00	6.00	6.00	6.00	4.00	4.00	4.00	4.00
Soybean meal (46% CP)	16.00	15.00	14.80	15.00	11.50	10.00	9.80	9.80
Rapeseed meal	10.00	10.00	10.00	10.00	10.50	10.00	10.00	10.00
Soybean oil	3.00	1.40	1.20	0.40	1.80	0.60	0.40	-
L-lysine HCL	0.30	0.30	0.30	0.28	0.25	0.28	0.28	0.28
DL-methionine	0.04	0.04	0.04	0.02	-	-	-	-
L-threonine	0.06	0.06	0.06	0.04	0.03	0.05	0.05	0.05
Calcium carbonate	1.10	0.94	0.95	0.95	1.02	0.92	0.90	0.90
Monocalcium phosphate	0.45	-	-	-	0.30	-	-	-
Sodium chloride	0.30	0.28	0.26	0.26	0.35	0.25	0.25	0.25
Vitamin-mineral premix^2^	0.25	0.25	0.25	0.25	0.25	0.25	0.25	0.25
Calculated nutrients, % unless otherwise stated								
Net energy, kcal/kg	2,380	2,330	2,325	2,292	2,356	2,315	2,306	2,280
Ash	5.10	4.44	4.36	4.47	4.71	4.19	4.14	4.32
Crude protein	17.85	17.78	17.72	17.80	16.44	16.10	16.04	15.98
Crude lignin	5.02	3.25	3.23	2.46	3.51	2.32	2.33	2.04
Crude fiber	4.43	4.45	4.44	4.47	4.30	4.24	4.21	4.20
Starch	37.7	39.8	40.0	40.3	41.4	43.9	43.8	44.1
Lysine (SID)	1.10 (0.95)	1.07 (0.93)	1.07 (0.93)	1.06 (0.92)	0.97 (0.83)	0.95 (0.81)	0.94 (0.81)	0.95 (0.81)
Methionine (SID)	0.33 (0.30)	0.33 (0.29)	0.33 (0.29)	0.32 (0.28)	0.28 (0.25)	0.27 (0.31)	0.27 (0.24)	0.27 (0.24)
Methionine + cysteine (SID)	0.68 (0.58)	0.66 (0.54)	0.66 (0.54)	0.65 (0.53)	0.63 (0.54)	0.61 (0.53)	0.61 (0.53)	0.61 (0.53)
Threonine (SID)	0.71 (0.60)	0.70 (0.58)	0.70 (0.58)	0.69 (0.57)	0.64 (0.52)	0.62 (0.50)	0.61 (0.49)	0.60 (0.48)
Tryptophan (SID)	0.21 (0.17)	0.20 (0.16)	0.20 (0.16)	0.20 (0.16)	0.19 (0.16)	0.19 (0.16)	0.19 (0.15)	0.19 (0.16)
Valine (SID)	0.85 (0.71)	0.82 (0.69)	0.82 (0.68)	0.81 (0.68)	0.80 (0.66)	0.77 (0.63)	0.76 (0.63)	0.77 (0.63)
Isoleucine (SID)	0.72 (0.65)	0.70 (0.63)	0.70 (0.62)	0.70 (0.62)	0.66 (0.56)	0.63 (0.53)	0.63 (0.53)	0.63 (0.53)
Leucine (SID)	1.29 (1.17)	1.26 (1.14)	1.26 (1.14)	1.26 (1.14)	1.19 (1.03)	1.14 (1.00)	1.13 (0.99)	1.13 (0.99)
Calcium	0.70	0.56	0.56	0.56	0.65	0.54	0.53	0.53
Phosphorus	0.55	0.43	0.42	0.42	0.51	0.43	0.43	0.43
ATTD phosphorus	0.24	0.14	0.13	0.13	0.23	0.15	0.15	0.14
Sodium	0.20	0.15	0.14	0.15	0.20	0.15	0.15	0.15
Phytate phosphorus	0.28	-	-	-	0.28	-	-	-

^1^Treatment details are given in Table 1.

^2^Provided per kilogram of diet: 4,800 IU of Vitamin A; 1,200 IU of Vitamin D3; 100.00 mg of Vitamin E; 1.20 mg of Vitamin K3; 0.80 mg of Vitamin B1; 2.40 mg of Vitamin B2; 0.80 mg of Vitamin B6; 16.00 μg of Vitamin B12; 12.00 mg of Niacinamide (niacin); 8.00 mg of Calcium-D-pantothenic acid; 50.00 mg of Betaine; 46.08 mg Choline chloride; 40.00 mg of Fe (sulfate); 10.00 mg of Cu (Cu-chelate), 32.00 mg of Mn (oxide); 48.00 mg of Zn (oxide); 0.80 mg of I; and 0.28 mg of Sodium selenate.

ATTD, apparent total tract digestible; NE, net energy; SID, standardized ileal digestible.

**Table 4.** Ingredient composition (as-fed basis) and calculated nutrient content of the experimental diets^1^ during finisher phase (exclusive of supplemental enzymes)

	Finisher (85 to 115 kg BW)			
	PC	NC1 + PhyG low	NC2 + PhyG high	NC3 + PhyG low + XB
Ingredient, % as is, unless otherwise stated				
Wheat	21.12	27.49	26.75	27.52
Corn	10.00	10.00	10.00	10.00
Barley	15.00	15.00	15.00	15.00
Rye	20.00	20.00	20.00	20.00
Wheat bran	10.00	10.00	10.00	10.00
Soybean meal (46% CP)	5.00	5.00	5.00	5.00
Rapeseed meal	14.50	12.50	11.50	11.00
Soybean oil	2.50	0.50	0.30	-
L-lysine HCL	0.23	0.23	0.23	0.23
DL-methionine	-	-	-	-
L-threonine	-	-	-	-
L-tryptophan	-	-	-	-
Calcium carbonate	1.00	0.83	0.77	0.80
Monocalcium phosphate	0.10	-	-	-
Sodium chloride	0.35	0.25	0.25	0.25
Vitamin-mineral premix^2^	0.20	0.20	0.20	0.20
Calculated nutrients, % unless otherwise stated				
Net energy, kcal/kg	2,309	2,268	2,260	2,231
Ash	4.60	4.12	4.05	4.15
Crude protein	15.2	14.9	14.8	14.8
Crude lignin	4.48	2.45	2.29	1.99
Crude fiber	4.76	4.47	4.52	4.47
Starch	39.8	43.2	43.2	43.7
Lysine (SID)	0.86 (0.71)	0.83 (0.69)	0.82 (0.68)	0.82 (0.68)
Methionine (SID)	0.26 (0.23)	0.26 (0.22)	0.25 (0.22)	0.25 (0.22)
Methionine + cysteine (SID)	0.60 (0.51)	0.59 (0.50)	0.58 (0.49)	0.57 (0.48)
Threonine (SID)	0.56 (0.43)	0.54 (0.42)	0.54 (0.41)	0.54 (0.40)
Tryptophan (SID)	0.18 (0.14)	0.17 (0.14)	0.17 (0.14)	0.17 (0.13)
Valine (SID)	0.73 (0.58)	0.71 (0.56)	0.70 (0.56)	0.71 (0.56)
Isoleucine (SID)	0.58 (0.47)	0.56 (0.46)	0.56 (0.46)	0.56 (0.46)
Leucine (SID)	1.05 (0.89)	1.03 (0.87)	1.03 (0.87)	1.02 (0.86)
Calcium	0.60	0.50	0.48	0.50
Phosphorus	0.49	0.46	0.46	0.46
ATTD phosphorus	0.20	0.17	0.17	0.17
Sodium	0.20	0.15	0.15	0.15
Phytate phosphorus	0.28	-	-	-

^1^Treatment details are given in Table 1.

^2^The composition of the vitamin-mineral premix is as listed in footnote 2 of Table 3. ATTD, apparent total tract digestible; NE, net energy; SID, standardized ileal digestible.

### Measurements and Sampling

Final diets were sampled for the analysis of dry matter, crude protein (**CP**), crude fat, crude fiber (**CF**), ash, nitrogen-free extract, phytate-P and enzyme activities. Pigs were weighed individually at the start of the experiment, weekly during the starter phases and at the end of each phase thereafter. Feed disappearance was recorded per pen at the end of each phase. Pens were inspected daily for pig mortality and signs of illness and dead animals were removed and individually weighed. These data were used to calculate ADG (based on individual pig weight), mortality-corrected average daily feed intake (**ADFI**, on a pen basis) and gain:feed per pen, for each phase and cumulatively. On the last day of the experiment, pigs were transferred to a slaughterhouse and eviscerated for the determination of carcass part weights (kg) and measurements including dressing percentage, lean meat percentage, loin meat length (mm) and backfat depth (mm).

### Chemical Analysis

Proximate analysis of feed samples was performed by SGS Analytics Germany GmbH, Jena, Germany according to the methods published by (VDLUFA 1976). Dry matter (**DM**) EC 152/2009, III; CP EG Dumas, DIN EN ISO 16634: 2016-11; crude fat (CL) EC 152/2009, III method B; CF EC 152/2009 III; ash EC 152/2009, III M. Phytase activities in feed were determined by Danisco Animal Nutrition Research Centre (Brabrand, Denmark) according to a modified version of the 2000.12 AOAC method according to Engelen et al. (2001). One phytase unit (**FTU**) was defined as the quantity of enzyme that released 1 µmol of inorganic orthophosphate from a 0.0051 mol/L sodium phytate substrate per minute at pH 5.5 at 37 °C. Phytate (IP_6_) in feed was analyzed at Danisco Animal Nutrition Research Centre (Brabrand, Denmark) using the HPLC method described by Christensen et al. (2020) modified from Skoglund et al. (1998). Xylanase analysis was conducted in duplicate at the Danisco Animal Nutrition Research Centre (Brabrand, Denmark), and reported as activity units as described by Romero et al. (2013). One xylanase unit was defined as the amount of enzyme that released 0.48 mmol of the reducing sugar xylose from wheat arabinoxylan per min at pH 4.2 and 50 °C. The β-glucanase activity was analyzed at the Danisco Animal Nutrition Research Centre (Brabrand, Denmark) using an internal colorimetric method. Briefly, a 100 mM acetate buffer (pH 5.0) was prepared. The stop solution was a 2% Tris buffer. Duplicate feed samples of 5 g were weighed, mixed with 50 mL of assay buffer. The standard enzyme was diluted to approximately 9 U/ml and added in increasing amounts to 6 blank feed samples. All samples were stirred for 10 min and then filtered, and 1 ml was added to test tubes. All samples were pre-incubated at 50 °C for 5 min. Beta-glucazyme tablets were then added to the incubated tubes, and the reaction was stopped after 30 min by adding the stop solution. Samples were centrifuged for 10 min at 3,500 rpm, and absorbance was measured at 590 nm. Enzyme activity was calculated against the standard curve response.

### Feed Costs and Carbon Footprint Calculations

Feed intake and body weight gain determinations were used to calculate the total cost of feed (in euros) per kilogram body weight gain during the entire wean-to-finish period, based on feed ingredient prices in Germany during October 2023. The cost of the supplemental enzymes was excluded from this analysis on the basis that these are variable and low relative to the cost of other ingredients in the diet. An estimate of the CFP of each of the treatment diets per kilogram of body weight gain during the entire wean-to-finish period was made using the FeedPrint database (FeedPrint NL, Wageningen University and Research, 2020). The tool calculates the CFP of feed raw materials during their complete life cycle, including crop production, processing of crop and animal products, compound feed production and utilization by the animal, including transport and storage between all steps of the production chain. It includes the CFP from land use and land use change.

### Statistical Analysis

For the analysis of gain:feed per phase and cumulatively during starter I and II and grower–finisher phases, pen was the experimental unit. The gain:feed during these phases was calculated based on total feed intake per pen and total BW gain per pen (including the weight of dead pigs), i.e., corrected for mortality. The ADG for these phases was calculated individually for each pig based on BW for each phase and ADFI was calculated by multiplying ADG with feed:gain for each phase (mortality-corrected). For the total experimental period (140 d, 7 to 115 kg BW), the differing number of replications used during the starter (I and II) and grower-finisher phases necessitated all performance measure calculations being made on an individual pig basis. For this period, the overall ADG was calculated as: (final BW-initial BW)/total no. of days (140), for each pig. The overall feed intake was calculated by adding together the feed intake of pigs during the starter phases with that of pigs during the grower-finisher period on a per pig basis; ADFI was then calculated as overall feed intake divided by the total number of d (140). Overall gain:feed was calculated as overall ADG divided by overall ADFI.

All data were analyzed by one-way ANOVA. Differences between treatment means were determined by Tukey’s HSD test. All statistical analyses were performed in JMP version 16.0 (SAS Institute, Inc., Cary, NC; JMP, 2022). A *P* value of < 0.05 was considered statistically significant.

## RESULTS

### Analyzed Nutrients and Enzyme Activity

Concentrations of analyzed nutrients and enzyme activities in the final diets are presented in Table 5. Diet analyses indicated that the intended concentrations of nutrients were present in all diets. Endogenous phytase activity in the unsupplemented PC diets was variable and quite high in grower II and finisher phase (467, 361, 685, 739 and 1,008 FTU/kg in starter I, starter II, grower I, grower II and finisher phase, respectively) but consistently lower than in the phytase-supplemented diets. Phytase activities were consistently higher in the ‘high’ than the respective ‘low’ dose phytase treatments, with good spacing achieved between the dose levels. Xylanase and β-glucanase activities in the NC3 + PhyG low + XB diets were acceptable to confirm that the target dose levels had been approximately met.

**Table 5.** Analyzed nutrient composition and enzyme activities in the treatment diets^1^, as-fed basis

	Dry matter, %	Crude protein, %	Crude fat, %	Crude fiber, %	Ash, %	Nitrogen-free extract, %	Phytate-P, %	Phytase, FTU/kg	Xylanase, XU/kg	β-glucanase, U/kg
Starter I (7 to 11 kg BW)										
PC	87.0	19.4	5.3	2.6	5.0	54.6	0.216	467	-	-
NC1 + PhyG low	86.2	18.6	4.4	2.7	4.3	56.2	-	1,615	-	-
NC2 + PhyG high	86.5	18.5	4.3	2.6	4.4	56.8	-	2,597	-	-
NC3 + PhyG low + XB	87.1	18.7	3.7	2.6	4.4	57.7	-	1,855	3,142	375
Starter II (11 to 25 kg BW)										
PC	89.5	21.2	4.7	4.0	5.2	54.4	0.274	361	-	-
NC1 + PhyG low	89.1	21.1	3.5	3.4	4.8	56.3	-	1,093	-	-
NC2 + PhyG high	89.5	20.6	3.6	3.2	5.2	57.0	-	2,625	-	-
NC3 + PhyG low + XB	89.3	20.8	3.2	3.5	4.7	57.1	-	1,620	2,780	317
Grower I (25 to 55 kg BW)										
PC	88.9	18.6	4.8	5.4	7.1	53.0	0.327	685	-	-
NC1 + PhyG low	89.4	18.7	2.9	5.0	4.9	57.9	-	1,051	-	-
NC2 + PhyG high	88.6	18.9	2.7	5.2	4.6	57.3	-	1,409	-	-
NC3 + PhyG low + XB	89.5	18.3	2.7	4.6	4.6	50.4	-	1,147	3,077	315
Grower II (55 to 85 kg BW)										
PC	88.2	16.9	4.9	4.5	4.6	57.3	0.308	739	-	-
NC1 + PhyG low	88.4	17.0	3.5	4.5	4.3	59.2	-	1,086	-	-
NC2 + PhyG high	88.3	16.8	3.1	4.3	4.2	59.8	-	1,347	-	-
NC3 + PhyG low + XB	88.0	16.7	2.5	4.6	4.3	60.0	-	984	2,915	316
Finisher (85 to 115 kg BW)										
PC	88.6	14.7	4.9	4.7	4.0	60.4	0.311	1,008	-	-
NC1 + PhyG low	88.3	14.2	3.7	4.2	3.7	62.5	-	1,268	-	-
NC2 + PhyG high	88.6	14.5	3.5	4.3	3.6	62.7	-	1,561	-	-
NC3 + PhyG low + XB	89.0	14.6	2.4	4.3	3.7	64.0	-	1,066	2,304	269

^1^Treatment details are given in Table 1.

BW, body weight.

### Growth Performance

The effect of treatment on growth performance by phase and cumulatively is presented in Tables 6 and 7, respectively.

**Table 6.** Growth performance of pigs fed a nutrient- and energy-reduced mixed-cereal diet supplemented with phytase (PhyG) without or with a xylanase–β-glucanase combination (XB); results by phase^1^

	PC	NC1 + PhyG low	NC2 + PhyG high	NC3 + PhyG low +XB	SEM	*P*—value
Initial body weight (d 0 on trial), kg/pig	7.19	7.18	7.18	7.18	-	-
Starter I (7 to 11 kg)						
Body weight at end, kg/pig	9.66	9.74	9.84	10.01	0.159	0.434
Average daily gain, g/pig/day	250	243	258	262	11.52	0.671
Average daily feed intake, g/pig/day	316	320	321	337	13.62	0.717
Gain:feed	0.79	0.77	0.80	0.79	0.007	0.064
Starter II (11 to 25 kg)						
Body weight at end, kg/pig	26.37^b^	26.70^ab^	26.81^ab^	28.19^a^	0.499	0.049
Average daily gain, g/pig/day	557^b^	573^ab^	564^b^	620^a^	13.62	0.006
Average daily feed intake, g/pig/day	837	847	849	897	20.03	0.153
Gain:feed	0.67^b^	0.68^ab^	0.66^b^	0.69^a^	0.005	0.001
Grower I (25 to 55 kg)						
Body weight at end, kg/pig	59.99^b^	62.59^ab^	63.16^ab^	65.10^a^	1.035	0.005
Average daily gain, g/pig/day	777^b^	835^ab^	847^a^	859^a^	15.24	0.002
Average daily feed intake, g/pig/day	1,704	1,704	1,761	1,802	35.22	0.148
Gain:feed	0.46^b^	0.49^a^	0.49^a^	0.48^a^	0.006	<0.001
Grower II (55 to 85 kg)						
Body weight at end, kg/pig	85.65	86.89	87.51	90.20	1.353	0.098
Average daily gain, g/pig/day	1,069	1,013	1,014	1,046	22.40	0.243
Average daily feed intake, g/pig/day	2,407	2,361	2,405	2,561	28.83	0.092
Gain:feed	0.45^a^	0.43^ab^	0.42^bc^	0.41^c^	0.005	<0.001
Finisher (85 to 115 kg)						
Body weight at end, kg/pig	114.49	114.81	115.64	116.69	1.656	0.775
Average daily gain, g/pig/day	1,030	997	1,005	952	21.27	0.085
Average daily feed intake, g/pig/day	2,747	2,800	2,828	2,725	65.11	0.664
Gain:feed	0.37	0.36	0.36	0.35	0.010	0.05

^1^Treatment details are given in Table 1.

**Table 7.** Cumulative growth performance of pigs fed a nutrient and energy reduced mixed cereal diet supplemented with phytase (PhyG) without or with a xylanase–β-glucanase combination (XB) from wean to finish^1^

	PC	NC1 + PhyG low	NC2 + PhyG high	NC3 + PhyG low + XB	SEM	*P*—value
Starter I and II phases (7 to 25 kg BW)						
Average daily gain, g/pig/day	418	424	426	457	10.91	0.063
Average daily feed intake, g/pig/day	603	607	611	639	15.37	0.345
Gain:feed	0.69^b^	0.70^b^	0.70^b^	0.72^a^	0.004	0.001
Grower finisher phases (25 to 115 kg BW)						
Average daily gain, g/pig/day	925	927	936	933	14.14	0.954
Average daily feed intake, g/pig/day	2,189	2,193	2,238	2,262	38.51	0.479
Gain:feed	0.42	0.43	0.42	0.41	0.004	0.121
Wean finish (7 to 115 kg BW)						
Average daily gain, g/pig/day	761	763	770	778	11.34	0.766
Average daily feed intake, g/pig/day	1,672	1,676	1,707	1,731	28.54	0.418
Gain:feed	0.45	0.46	0.45	0.45	0.003	0.309

^1^Treatment details are given in Table 1.

Considering the total experimental period (140 d), pigs fed the PC attained a final average BW of 114.4 kg. The final BW of pigs fed the enzyme-supplemented, nutrient-reduced diets did not differ from this (114.8, 115.6 and 116.7 kg in NC1 + PhyG low, NC2 + PhyG high and NC3 + PhyG low + XB, respectively, *P* > 0.05). The overall ADG, ADFI and gain:feed also did not differ in the enzyme-supplemented, nutrient-reduced diets compared to the PC.

During starter I phase (7 to 11 kg BW; Table 6), BW, ADG and ADFI were maintained in all enzyme-supplemented diets at levels that were not different from those achieved by the PC. The gain:feed tended to differ by treatment (*P* = 0.064) but the value of the treatment means did not provide a clear indication of a difference in the NC treatments relative to the PC. During starter II phase (11 to 25 kg BW), ADFI did not differ among treatments, whereas ADG, BW and gain:feed were all increased compared with the PC in NC3 + PhyG low + XB (ADG: + 63 g/pig or 11.3%, BW: + 1.82 kg/pig or 6.9%, gain:feed: + 0.02 or 2.9%, respectively; *P* < 0.05) and maintained vs. PC in NC1 + PhyG low and NC2 + PhyG high. During this phase, the combination of PhyG at low dose with XB in NC3 resulted in a greater ADG than PhyG at high dose without XB in NC2 (+ 56 g/pig or 9.9%; *P* < 0.05). For the cumulative period covering starter I and II phases, gain:feed was increased in NC3 + PhyG low + XB vs. NC2 + PhyG high or PC. During grower I (25 to 55 kg), ADFI did not differ among treatments whereas BW was maintained at a level not different from that achieved by the PC in NC1 + PhyG low and NC2 + PhyG high but was greater than PC in NC3 + PhyG low + XB (+ 5.11 kg/pig or + 8.5%; *P* < 0.05); ADG was greater in both NC2 + PhyG high and NC3 + PhyG low + XB vs. PC (*P* < 0.05) and maintained vs. PC in NC1 + PhyG low. During grower I, gain:feed was increased (*P* < 0.05) in all enzyme-supplemented treatments vs. PC. During grower II phase (55 to 85 kg), BW, ADG and ADFI did not differ among treatments. During finisher phase and the cumulative grower-finisher period, growth performance measures were maintained in all enzyme-supplemented diets to a levelnot different from that achieved by the PC.

### Feed Costs and Carbon Footprint

**Figure 1.**
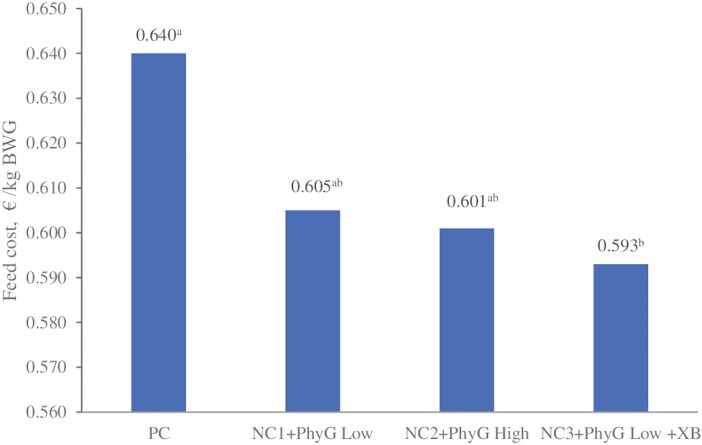
**Comparison of the total calculated feed costs** ^**1**^ **of the experimental diets** ^**2**^ **per kilogram of body weight gain (BWG) during wean to finish.** ^1^Feed costs were calculated based on market prices for feed ingredients in Germany in October 2023, exclusive of the costs of enzymes. ^2^Treatment details are given in Table 1. ^a,b^Bars bearing different superscript letters are significantly different at *P* < 0.05; ANOVA *P*-value = 0.037. BWG, body weight gain.

A comparison of the total estimated feed costs per kg body weight gain, for the entire wean-to-finish period, based on ingredient prices in October 2023, is presented in Figure 1. The calculations were exclusive of the cost of the enzymes which varies over time and is low relative to the cost of other ingredients. Feed costs were significantly reduced (*P* < 0.05) in NC3 + PhyG low + XB compared with the PC [by 0.047 €/kg BW gain (BWG), equivalent to 7.3%] and numerically reduced in NC1 + PhyG low and NC2 + PhyG high vs. PC (by 0.035 and 0.039 €/kg BWG, equivalent to 5.5% and 6.1%, respectively).

**Figure 2.**
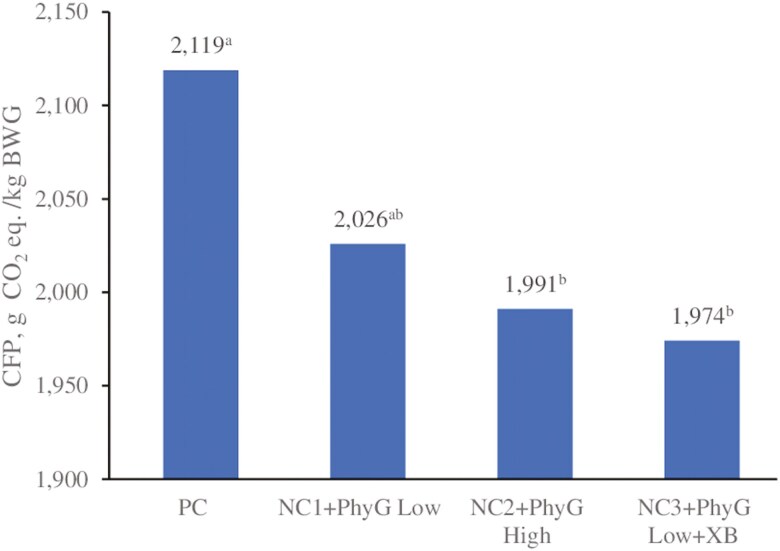
**Comparison of the calculated carbon footprint (CFP)** ^**1**^ **of the experimental diets** ^**2**^ **per kilogram of body weight gain (BWG) during wean to finish.** ^1^The total carbon footprint includes the CFP from fossil fuels and from land use change. Calculations made using Wageningen Feedprint NL software (Feedprint NL, 2020). ^2^Treatment details are given in Table 1. ^a,b^Bars bearing different superscript letters are significantly different at *P* < 0.05; ANOVA *P*-value = 0.008.

An estimation of the CFP of the treatment diets per kilogram of BW gain is presented in Figure 2. The CFP was significantly reduced in NC2 + PhyG high and NC3 + PhyG low + XB compared with the PC (by 128 and 145 g CO_2_ equivalents per kilogram of BWG, respectively, equivalent to reductions of 6.0% and 6.8%; *P* < 0.05).

### Carcass Characteristics

All carcass measures in the nutrient-reduced, enzyme-supplemented diets were maintained at levels that were not different (*P* > 0.05) from those achieved by the PC (Table 8). The average values across all treatments were 78.4% and 62.1% for dressing and lean meat percentage, respectively, and 70.3 and 12.2 mm for loin meat and backfat thickness, respectively.

**Table 8.** Carcass characteristics of pigs fed a nutrient and energy-reduced mixed cereal diet supplemented with phytase (PhyG) without or with a xylanase–β-glucanase combination (XB) from wean to finish^1^

	PC	NC1 + PhyG Llow	NC2 + PhyG High	NC3 + PhyG Low + XB	SEM	*P*—value
Carcass weight, kg	94.6	93.9	93.6	94.5	1.12	0.923
Dressing, %	77.9	78.6	78.3	78.7	0.39	0.548
Lean meat, %	61.4	62.3	62.5	62.1	0.31	0.101
Loin meat, mm	70.0	70.2	70.2	70.9	0.94	0.926
Backfat, mm	13.0	11.9	11.7	12.2	0.35	0.109

^1^Treatment details are given in Table 1.

## DISCUSSION

The nutrient and enzyme analysis of the treatment diets indicated that the applied nutrient reductions and enzyme supplementation levels had been achieved approximately as intended. The energy reduction was validated by the lower analyzed fat content in the test diets relative to the PC. Analyzed phytase activities in the PC diets were variable and, in some cases, quite high (467, 361, 685, 739 and 1,008 FTU/kg, in starter I, II, grower I, II and finisher phase, respectively). This is likely because the basal diet contained a substantial content of wheat, barley and rye (together making up 36% to 57% of the diet, across phases). These cereals, and in particular rye, have a considerably higher content of native phytase than corn (Delia et al., 2011) and their addition to the diet can by itself increase the digestibility of P in other cereal ingredients such as corn and soybean meal (Poulsen et al., 2019; Archs Toledo et al., 2020), separate to the effect of added microbial phytase (Zimmerman et al., 2003). However, the native phytase has a higher pH optimum and is therefore less effective than microbial phytase for increasing P digestibility (Weremko et al., 2001). The very high analyzed phytase activity in the PC grower II and finisher diets seems unlikely and could have been due to analytical issues. However, as the ingredient composition of the PC and test diets was similar, it is likely that the test diets would also have contained a high level of intrinsic phytase. Irrespective of this, the presence of added phytase in the phytase-supplemented diets was confirmed by the higher phytase recoveries from these diets compared with the respective PC, and there was a clear dose difference between 'low' and 'high' phytase treatments. Based on this, and given that intrinsic phytase is well known to have a higher pH optimum (and therefore is less effective in vivo) than added microbial phytase, it is considered unlikely that the intrinsic phytase would have confounded the comparisons among treatments.

Pigs fed the nutritionally adequate PC diet attained overall performance levels consistent with expectations under European production standards; average BW at slaughter (114 kg) was within the range (110 to 115 kg) that is considered optimal for commercial pig production in Germany where the experiment was conducted, and was reached within 140 d, comparable with commercial practice. On this basis, it was concluded that the PC formed an appropriate benchmark against which to evaluate the capacity of the supplemental enzymes to compensate for the nutrient and energy reductions applied to the NC diets.

Growth performance outcomes were maintained in NC1 + PhyG low and NC2 + PhyG high at a level that was not different from, or was improved, compared with the PC, for all measures during all phases, except gain:feed in grower II phase, during which the average response in the enzyme-supplemented diets was in a different direction to that observed in grower I. The reason for this is unclear. However, when the overall experimental period was considered, the results indicated that the supplemental phytase activity compensated for any negative effect of the nutrient and energy reductions on performance, from wean to finish, resulting in overall growth performance that was not different form the PC. The two possible contributors to this effect are 1) increased feed (and thereby nutrient) intake, which could have been a response to the added phytase, and 2) increased feed efficiency and utilization of nutrients due to the nutrient “releasing” activity of the added phytase. Increased feed intake in response to PhyG added to a nutrient-reduced diet has been observed previously (Velayudhan et al., 2021; pooled data across 500 and 1,000 FTU/kg dose levels compared with the response to the NC without supplemental phytase). The absence of a stand-alone NC in the present experiment precludes any assessment of whether PhyG increased feed intake relative to no PhyG but the data show that feed intake in the PhyG-supplemented treatments was not raised above the level of the PC during any individual phase or overall. It follows that if feed intake and overall performance measures (ADG, final BW, gain:feed) did not differ from the PC and yet the nutrient and energy content of the feed had been reduced, it was most likely the added phytase that increased nutrient and energy availability enabling growth performance to be maintained at a level not different from the PC. This is supported by the existing literature on the in vitro and in vivo mode of action of PhyG that describes its extensive hydrolysis of IP_6_ in the digesta to low IP-esters, improving IP_6_ digestibility (Christensen et al., 2020). It is also supported by the observation that gain:feed was improved beyond the level of the PC in NC1 + PhyG low and NC2 + PhyG high treatments during grower I phase suggesting greater feed efficiency in the presence of the phytase despite the nutrient and energy reductions. In growing pigs (23 to 55 kg BW) fed nutrient- and energy-reduced diets, Velayudhan et al. (2021) observed a positive linear relationship between PhyG dose level (up to 1,000 FTU/kg) and feed efficiency (feed conversion ratio) and PhyG also increased gain:feed in pigs of 12 to 25 kg BW in an experiment by Espinosa et al. (2021). In a separate experiment by the latter authors, the same phytase supplemented within the range 250 to 4,000 FTU/kg resulted in dose-dependent increases in the apparent ileal digestibility of P, Ca, energy, AA and Na in pigs of starting weight ~17 kg (Espinosa et al., 2022). These data provide a basis for the thesis that, in the present experiment, increased nutrient availability and utilization due to the activity of the supplemental PhyG was responsible for the maintenance of growth performance to a level not different from the PC in the PhyG-supplemented diets. The data also support that the response to PhyG was related to dose: PhyG application at a 'high' dose level to the more severely nutrient- and energy-reduced NC2 diet achieved growth performance outcomes that were not significantly different (during any phase or overall) to those achieved by its application at a 'low' dose level to the less severely nutrient- and energy-reduced NC1. The improvement in ADG beyond the level of the PC in NC2 + PhyG high in grower I phase indicates that the nutrient and energy reductions applied to this treatment were overly conservative during this single phase. However, over the entire wean-to-finish period, this effect was not evident. Hence, it is concluded that the applied nutrient and energy reductions were appropriate when viewed over the whole wean-to-finish period.

The addition of the xylanase–β-glucanase combination to NC3 containing PhyG supplementation at ‘low’ dose, with additional reduction of NE and digestible AA vs. NC2 and NC1, was also effective in compensating for the applied nutrient and energy reductions. This was evidenced by growth performance (all measures) being not different from, or improved, in NC3 + PhyG low + XB vs. PC, NC1 + PhyG low and NC2 + PhyG high, for the overall wean-to-finish period. Given the further 37.5 kcal/kg NE and digestible AA reduction in diet NC3 + PhyG low + XB vs. NC1 + PhyG low with the same level of phytase applied and yet overall growth performance in both treatments was not different from the PC, the implication is that there was some beneficial effect of the xylanase–β-glucanase beyond that of the phytase. Kiarie et al. (2012) observed increased total tract digestibility of energy in grower pigs fed reduced-nutrient diets supplemented with the xylanase–β-glucanase at the same dose level applied in the present experiment (2,440 U/kg xylanase and 304 U/kg β-glucanase) that led to improved weight gain compared with an NC. In that experiment, all diets contained phytase as a background enzyme (dosed at 500 FTU/kg) so the observed improvements were attributable to the xylanase–β-glucanase over and above the effect of the phytase.

The carcass characteristics data demonstrated that the improvements in live weight gain and body mass in the enzyme-supplemented diets (that resulted in growth performance outcomes that were not different from the PC) were carried through into favorable production outcomes. This included the percentages of lean and loin meat, backfat and dressing, that are key characteristics of value at the point of sale. The enzyme-supplemented treatments performed equally in this regard. However, the associated feed costs and estimated CFP of the diets per kg of weight gain were not equal across treatments. The reduced total feed costs and reduced CFP of all 3 enzyme-supplemented treatments vs. PC will be of interest to today’s producers who are focused on reducing the overall costs of production and increasing sustainability, without comprising growth performance objectives and production outcomes. The use of exogenous enzymes to enhance nutrient availability from more challenging diets containing multiple cereal types in addition to fibrous co-products can be useful in this regard.

In conclusion, the application of matrix values for Ca, energy, digestible AA and Na to a mixed-cereal diet based on wheat, corn, barley, rye and soybean meal, without added inorganic P, in combination with addition of a consensus bacterial 6-phytase in a phased dosing regimen, maintained growth performance and carcass characteristics of pigs at a level that was not different from that achieved by a nutritionally adequate unsupplemented diet, from wean to finish (7 to 115 kg). Using a higher phytase dosing regimen enabled the use of greater nutrient and energy reductions without affecting growth performance when compared with a lower dosing regimen applied with lower nutrient and energy reductions, and also delivered a further reduction in feed cost and in CFP per kg of BW gain. When a xylanase–β-glucanase combination was added on top of the phytase (applied at the lower dosing regimen), with an additional 37.5 kcal/kg reduction in NE and further reduction in digestible AA, growth performance was also maintained at a level not different from the PC. These results confirm the appropriateness of the applied matrix values for PhyG, with or without xylanase-β-glucanase, in a mixed-cereal diet without added inorganic P. The reductions in total feed costs and in CFP per kg BW gain enabled by the phytase or phytase and xylanase–β-glucanase were 0.035 to 0.047 €/kg BWG (5.5% to 7.3%) and 194 to 222 g CO_2_ equivalents/kg BWG, respectively, over the entire wean-to-finish period.
